# Selective PPARα Modulator Pemafibrate Ameliorates Metabolic Dysfunction-Associated Steatotic Liver Disease Through Regulation of Leptin Signaling and Remodeling of Gut Microbiota

**DOI:** 10.3390/ijms27177611

**Published:** 2026-08-25

**Authors:** Koji Yamamoto, Masaru Baba, Akinori Kubo, Ren Yamada, Akihisa Nakamura, Kenichi Morikawa, Masatsugu Ohara, Masato Nakai, Takuya Sho, Goki Suda, Koji Ogawa, Ken Furuya, Naoya Sakamoto

**Affiliations:** 1 Department of Gastroenterology and Hepatology, Hokkaido University Faculty of Medicine and Graduate School of Medicine, Sapporo 060-8648, Japan; 2 Department of Physiology, Asahikawa Medical University, Asahikawa 078-8510, Japan; 3 Department of Gastroenterology, JCHO Hokkaido Hospital, Sapporo 062-8618, Japan; 4 Keijinkai Maruyama Clinic, Sapporo 064-0820, Japan; 5 Department of Gastroenterology, Sapporo City General Hospital, Sapporo 060-8604, Japan; 6 Department of Gastroenterology, Japanese Red Cross Asahikawa Hospital, Asahikawa 070-8530, Japan; 7 Keimei Clinic, Sapporo 064-0913, Japan; 8 Department of Gastroenterology, NTT Medical Center Sapporo Hospital, Sapporo 060-0061, Japan

**Keywords:** leptin, MASLD, pemafibrate, gut–liver axis, gut microbiota

## Abstract

Metabolic dysfunction-associated steatotic liver disease (MASLD) is one of the leading causes of chronic liver disease worldwide, and effective pharmacological therapies remain limited. Pemafibrate (Pema), a selective peroxisome proliferator-activated receptor alpha (PPARα) modulator (SPPARMα), has shown promising therapeutic potential in patients with MASLD and hypertriglyceridemia; however, its underlying mechanisms remain incompletely understood. Here, we investigated the therapeutic effects and molecular mechanisms of Pema using in vitro and in vivo MASLD models and evaluated its clinical relevance in patients with MASLD. In a choline-deficient, L-amino acid-defined, high-fat diet (CDAHFD)-induced MASLD mouse model, Pema dose-dependently ameliorated hepatic steatosis and fibrosis and significantly suppressed the hepatic expression of inflammatory and fibrogenic genes, including TLR4, TNFα, αSMA, and Col1A1. Pema also favorably altered the gut microbiota by increasing the abundance of the phylum *Verrucomicrobia*, suggesting modulation of the gut–liver axis. In differentiated 3T3-L1 adipocyte-like cells, Pema induced PPARα expression, inhibited insulin-mediated EGR-1 induction, and significantly reduced leptin secretion. Consistent with these findings, serum insulin and leptin levels, as well as the hepatic accumulation of both molecules, were markedly decreased in Pema-treated MASLD mice. Furthermore, in a clinical cohort of 192 patients with MASLD and hypertriglyceridemia, long-term Pema treatment significantly improved liver-related biochemical parameters, insulin resistance, surrogate markers of liver fibrosis, and serum leptin levels. Collectively, these findings demonstrate that Pema attenuates MASLD progression by suppressing leptin-mediated metabolic and inflammatory signaling while improving the gut microenvironment. These results highlight SPPARMα as a promising therapeutic strategy for MASLD and support further clinical investigation of Pema as a disease-modifying treatment targeting the gut–liver axis.

## 1. Introduction

The global burden of viral hepatopathies has declined significantly in recent years, largely due to breakthroughs in antiviral treatments, such as nucleos(t)ide analogs for hepatitis B [1,2,3] and direct-acting agents for hepatitis C [4,5,6]. As a result of this epidemiological shift, metabolic dysfunction-associated steatotic liver disease (MASLD), previously termed nonalcoholic fatty liver disease (NAFLD), has emerged as a predominant chronic liver condition worldwide and a primary driver of non-viral cirrhosis and hepatocellular carcinoma [7]. Clinically, an MASLD diagnosis requires the identification of hepatic fat accumulation via imaging techniques or histological assessment, coupled with the presence of cardiometabolic risk factors. Driven by the expanding global epidemics of metabolic syndrome and obesity, the incidence of MASLD is surging rapidly, posing a severe threat to public health [8]. MASLD manifests across a clinical continuum. It begins with isolated fat accumulation, designated as metabolic dysfunction-associated steatotic liver. However, it can advance to metabolic dysfunction-associated steatohepatitis (MASH), a more severe phenotype defined by hepatocyte injury, inflammatory infiltration, and fibrogenesis. If MASLD is left undiscovered, this chronic inflammatory and fibrotic state can culminate in end-stage complications, including cirrhosis, hepatic failure, hepatocellular carcinoma, and increased liver-related mortality. Current epidemiological estimates suggest that MASLD affects roughly 25% of the global population, with its prevalence expected to climb continually [9]. Predictive models anticipate a substantial expansion in the number of MASLD and MASH patients within the United States over the next decade. A parallel trend is evident in Japan, where MASLD prevalence has nearly tripled over the last twenty years, and the clinical burden of both MASLD and MASH is forecasted to escalate further in the near future [10,11,12].

Although lifestyle modification through dietary intervention and regular physical activity can improve hepatic steatosis, advanced MASH with cirrhosis often requires liver transplantation because effective treatment options remain limited. Consequently, MASLD-related cirrhosis and hepatocellular carcinoma have become major causes of liver-related mortality in Japan and many other countries. In addition to lifestyle modification, therapeutic approaches currently include antidiabetic agents, lipid-lowering drugs, vitamin E supplementation, and bariatric surgery for selected patients. Numerous investigational agents targeting diverse molecular pathways are currently being evaluated in clinical trials; however, many candidates have failed to demonstrate sufficient efficacy or safety. Among these emerging therapeutic strategies, peroxisome proliferator-activated receptor (PPAR) agonists have attracted considerable attention because of their central role in regulating lipid metabolism and energy homeostasis. Despite the growing disease burden, pharmacological treatment options for MASLD remain limited [13,14,15,16]. Resmetirom and semaglutide [17] have recently been approved for the treatment of MASH; however, their therapeutic effects are not sufficient for all patients, and additional pharmacological approaches targeting lipid metabolism and hepatic inflammation remain necessary. Peroxisome proliferator-activated receptor α (PPARα) is a key regulator of hepatic lipid metabolism, suggesting that selective PPARα modulation may represent an alternative therapeutic strategy for MASLD/MASH.

Pemafibrate (Pema) is a novel selective PPARα modulator (SPPARMα) with high receptor selectivity and potent transcriptional activity, resulting in an improved safety profile compared with conventional fibrates. Activation of PPARα enhances hepatic fatty acid β-oxidation and regulates multiple genes involved in lipid metabolism, thereby reducing hepatic lipid accumulation [18,19]. Clinical studies conducted in Japan have demonstrated that Pema improves liver enzyme levels in patients with hypertriglyceridemia accompanied by liver dysfunction, and exploratory studies have suggested beneficial effects in patients with MASLD [20,21]. Furthermore, several reports have indicated that Pema improves insulin resistance [22,23]. Nevertheless, the molecular mechanisms responsible for these beneficial effects, particularly those linking lipid metabolism, adipokine regulation, and the gut–liver axis, remain incompletely understood.

To address these knowledge gaps, we investigated the therapeutic mechanisms of Pema using complementary in vitro and in vivo models of MASLD. In addition, we evaluated the clinical relevance of these findings in a cohort of patients with MASLD and hypertriglyceridemia. Our study focused on the regulation of leptin signaling and alterations in the gut microbiota as potential mechanisms underlying the therapeutic effects of SPPARMα in MASLD. The present study provides novel mechanistic insights by integrating in vitro experiments, diet-induced MASLD mouse models, gut microbiota analyses, leptin biology, and clinical validation.

## 2. Results

### 2.1. Baseline Characteristics of the Study Cohort

The demographic and clinical profiles of the 192 enrolled subjects are detailed in Table 1. Relative to baseline measurements, a one-year course of Pema therapy led to substantial reductions in serum levels of aspartate aminotransferase (AST), alanine aminotransferase (ALT), γ-glutamyl transferase (GGT), triglycerides (TG), low-density lipoprotein (LDL) cholesterol, and Wisteria floribunda agglutinin-positive Mac-2 binding protein (M2BPGi). Furthermore, following two years of intervention, subjects exhibited marked improvements in their platelet counts, HOMA-IR (a surrogate marker for insulin resistance), FIB-4 scores, and liver stiffness measurements (LSM).

### 2.2. Pema Mitigates Hepatic Steatosis and Fibrosis in a CDAHFD Mouse Model

**Figure 1:**
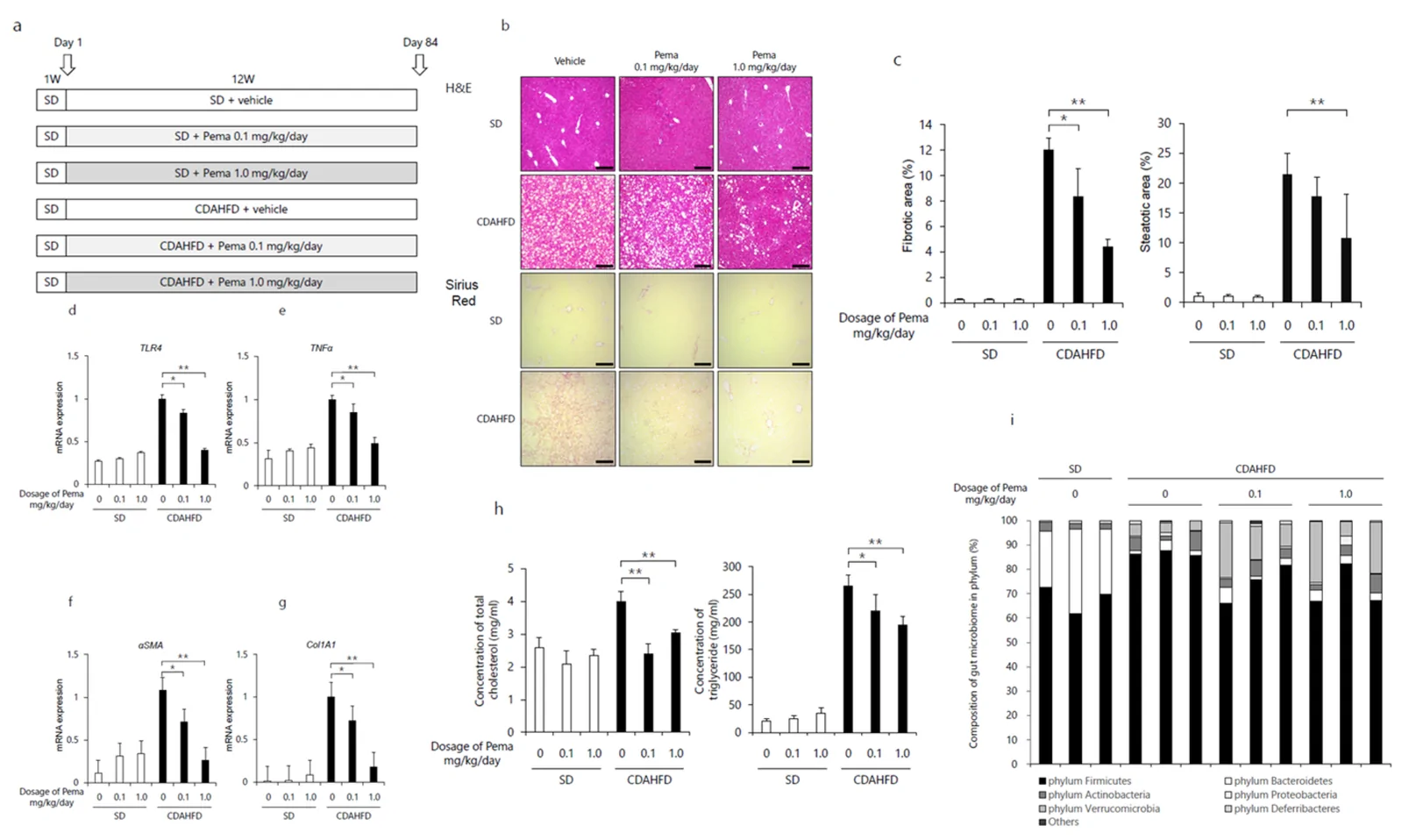
Analysis of C57BL/6J mice fed with SD and CDAHFD and receiving pemafibrate. (**a**) Experimental methods. Mice in all groups were fed SD for the first week, after which they were fed SD or CDAHFD for 12 weeks with administration of different doses of Pema (vehicle, 0.1 mg or 1 mg/kg/day) suspended in 0.5% MC using an oral sonde. (**b**) Representative photomicrographs of liver sections of mice fed with SD and CDAHFD with administration of Pema stained with H&E and Sirius Red at 12 weeks (10× magnification, scale bars = 250 μm). (**c**) Fibrotic and steatotic areas estimated using quantitative analysis were consistent with histological findings (n = 5 per group). Fold changes in hepatic mRNA expression of TLR4 (**d**), TNFα (**e**), αSMA (**f**), and Col1A1 (**g**) in mice fed with SD and CDAHFD with administration of Pema for 12 weeks (n = 8 per group). (**h**) Concentrations of total cholesterol and triglyceride in the livers of mice fed with SD and CDAHFD with administration of Pema for 12 weeks (n = 8 per group). (**i**) Representative composition of the gut microbiome (phylum) of mice fed with SD and CDAHFD (n = 3 per group) with administration of Pema at 12 weeks. Data are presented as means ± standard deviation. * *p* < 0.05, ** *p* < 0.01. αSMA, α smooth muscle actin; CDAHFD, choline-deficient, L-amino acid-defined, high-fat diet; Col1A1, collagen 1A1; H&E, hematoxylin and eosin; MC, methyl cellulose; Pema, pemafibrate; SD, standard diet; TLR4, Toll-like receptor 4; TNFα, tumor necrosis factor α.

Figure 1b presents the hepatic histological findings of mice maintained on either a standard diet (SD) or a choline-deficient, L-amino acid-defined, high-fat diet (CDAHFD), following 12 weeks of daily Pema administration (0, 0.1, or 1 mg/kg/day). Sirius Red staining demonstrated that Pema dose-dependently attenuated liver fibrosis in the CDAHFD-fed cohort. Additionally, hematoxylin and eosin (H&E) staining indicated that the highest dose of Pema (1 mg/kg/day) successfully alleviated hepatic fat accumulation. In contrast, SD-fed mice displayed no signs of fibrotic progression or steatotic development (Figure 1b,c).

### 2.3. Pema Exerts Anti-Inflammatory Effects in the Livers of CDAHFD-Fed Mice

Hepatic transcription levels of Toll-like receptor 4 (TLR4) (Figure 1d), tumor necrosis factor-alpha (TNF-α) (Figure 1e), alpha-smooth muscle actin (α-SMA) (Figure 1f), and collagen type I alpha 1 chain (Col1a1) (Figure 1g) were notably elevated in the CDAHFD group relative to the SD controls. Treatment with Pema effectively downregulated the expression of these pro-inflammatory and profibrogenic genes in a dose-proportional manner, indicating that Pema successfully represses hepatic inflammation triggered by the CDAHFD.

### 2.4. Impact of Pema on Hepatic Lipid Accumulation in CDAHFD Mice

To further elucidate Pema’s influence on hepatic lipid profiles in the CDAHFD model, intrahepatic total cholesterol and triglyceride (TG) concentrations were quantified (Figure 1h). Mice consuming the CDAHFD without Pema exhibited a profound elevation in both cholesterol and TG stores. Conversely, the introduction of Pema demonstrated a strong tendency to lower these lipid parameters within the livers of the affected mice.

### 2.5. Pema Alters the Intestinal Microbiome in CDAHFD-Fed Mice

Fecal microbiota profiling was conducted for both diet groups at the 12-week mark (Figure 1i). Relative to the SD animals, the CDAHFD control group demonstrated a significant microbial shift characterized by enriched Firmicutes and depleted Bacteroidetes populations (n = 8 per group, *p* < 0.01). Interestingly, Pema supplementation in the CDAHFD mice led to a dose-dependent expansion of the phylum *Verrucomicrobia* when compared to the SD controls (n = 8 per group, *p* < 0.01) (Table S1).

### 2.6. Pema Modulates Leptin Secretion in Differentiated Adipocyte-like Cells

**Figure 2:**
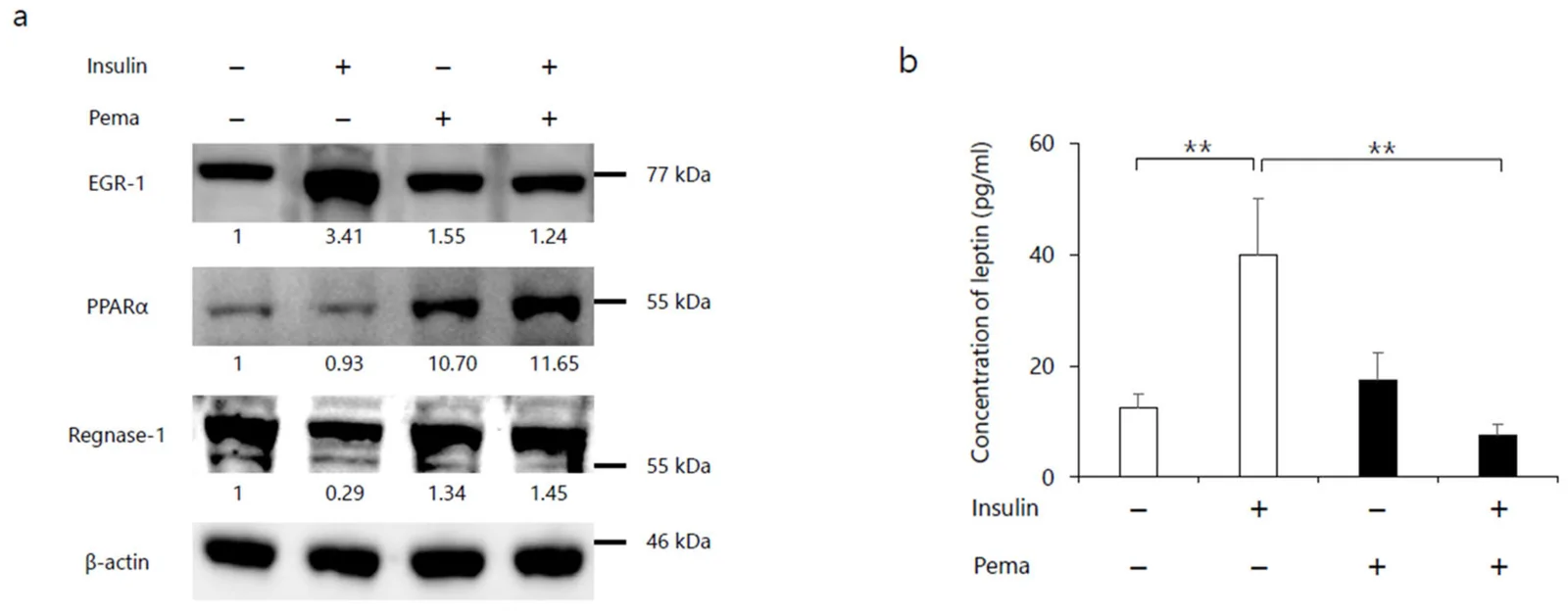
Pemafibrate suppresses leptin secretion from adipocyte-like cells. (**a**) Adipocyte-like cells differentiated from 3T3-L1 cells were treated with insulin and pemafibrate for 24 h. Cell lysates were separated by 15% sodium dodecyl sulfate–polyacrylamide gel electrophoresis, followed by immunoblotting using mAbs 44D5, ab126285, MABF936, and AC-15 against EGR-1, PPARα, regnase-1, and β-actin, respectively. Molecular weight standards are presented on the right. Densitometry analysis was performed, and relative ratio are presented under each band as numbers normalized to β-actin. (**b**) The concentration of leptin in the culture supernatant of adipocyte-like cells was measured. ** *p* < 0.01. EGR-1, early growth response protein 1; PPARα, peroxisome proliferator-activated receptor-α.

We utilized an in vitro culture system to investigate Pema’s influence on adipocyte-derived leptin release. Using 3T3-L1-derived adipocyte-like cells, we assessed the expression of EGR-1 (a key driver of insulin-stimulated leptin production [24,25]), its regulator regnase-1 (Reg-1), and PPAR-α. Treatment with insulin alone resulted in the downregulation of Reg-1 and the concurrent upregulation of EGR-1. Meanwhile, isolated Pema treatment stimulated PPAR-α expression. Notably, the simultaneous application of Pema blunted the insulin-driven induction of EGR-1 (Figure 2a). Subsequent quantification of leptin in the culture media confirmed that Pema effectively curtailed insulin-mediated leptin secretion (Figure 2b).

### 2.7. Pema Reduces Circulating and Hepatic Levels of Insulin and Leptin In Vivo

**Figure 3:**
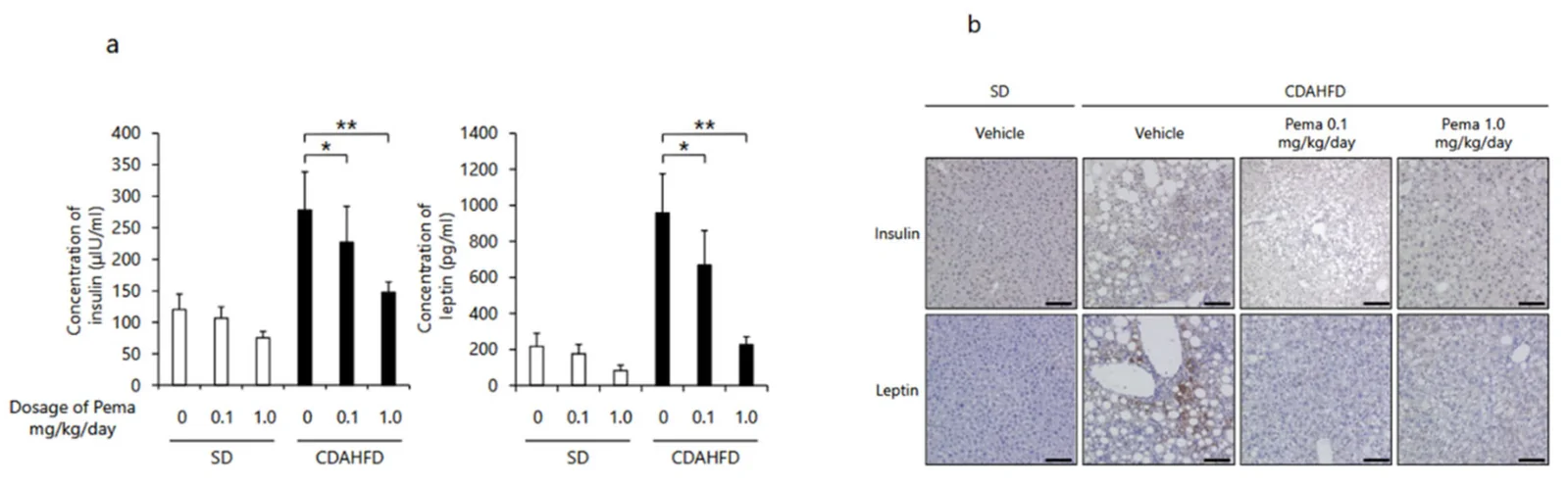
Insulin and leptin levels of C57BL/6J mice fed with SD and CDAHFD with administration of pemafibrate. (**a**) Serum insulin and leptin levels in C57BL/6J mice fed with SD and CDAHFD by administration of pemafibrate for 12 weeks. (**b**) Representative immunohistochemical (IHC) microscopic images of insulin and leptin accumulation in the liver at 12 weeks (10× magnification, scale bars = 200 μm). Data are presented as means ± standard deviation (SD). * *p* < 0.05, ** *p* < 0.01. CDAHFD, choline-deficient, L-amino acid-defined, high-fat diet; SD, standard diet.

Circulating insulin and leptin concentrations were significantly elevated in untreated CDAHFD mice relative to those on the standard diet (Figure 3a). Administration of Pema to the CDAHFD group yielded a dose-dependent reduction in these serum hormone levels. Immunohistochemical (IHC) analysis revealed substantial intrahepatic accumulation of both insulin and leptin in the CDAHFD controls, a phenomenon absent in the SD cohort (Figure 3b). Consistent with the serum findings, Pema treatment progressively diminished the hepatic pooling of insulin and leptin in a dose-dependent fashion.

### 2.8. Pema Decreases Serum Leptin in Patients with MASLD and Hypertriglyceridemia

Finally, we evaluated circulating leptin concentrations in a clinical cohort diagnosed with metabolic dysfunction-associated steatotic liver disease (MASLD) complicated by hypertriglyceridemia, both before and after therapy. Following Pema intervention, there was a statistically significant decline in serum leptin (*p* = 0.02) (Table 1). To further investigate this relationship, correlation analyses were performed in the 95 patients whose serum leptin levels were available (Table 2). We found that serum leptin levels before Pema administration showed positive correlations with BMI (r = 0.262, *p* = 0.01), AST (r = 0.265, *p* < 0.01), ALT (r = 0.252, *p* = 0.01), HDL cholesterol (r = 0.256, *p* = 0.01) and HOMA-IR (r = 0.393, *p* < 0.01), respectively. Among these parameters, AST (r = −0.256, *p* = 0.01), ALT (r = −0.224, *p* = 0.03) and HOMA-IR (r = −0.282, *p* = 0.01) showed negative correlations with serum leptin reduction in MAFLD patients receiving Pema. These results indicate that AST, ALT and HOMA-IR might be predictive markers for the reduction in serum leptin levels in MAFLD patients treated with Pema. However, we need to increase the number of cases to prove these correlations.

## 3. Discussion

The present study provides translational evidence that the selective PPARα modulator Pema exerts therapeutic effects in MASLD through regulation of leptin signaling. By integrating in vitro experiments, a diet-induced MASLD mouse model, and clinical observations from patients with MASLD and hypertriglyceridemia, we consistently demonstrated that Pema reduced leptin production, attenuated hepatic inflammation and fibrosis, and improved metabolic abnormalities. These findings suggest that suppression of leptin-mediated disease progression represents an important mechanism underlying the therapeutic effects of SPPARMα in MASLD.

Leptin is a key adipokine that plays a central role in maintaining systemic energy homeostasis by regulating appetite, energy expenditure, glucose metabolism, and lipid utilization [24]. Under physiological conditions, leptin enhances insulin sensitivity by limiting excessive food intake and promoting fatty acid oxidation [25,26]. However, obesity and MASLD are frequently accompanied by hyperleptinemia and leptin resistance, resulting in impaired metabolic regulation despite elevated circulating leptin concentrations. Persistent leptin signaling has also been implicated in hepatic inflammation and fibrogenesis, suggesting that dysregulated leptin signaling contributes not only to metabolic dysfunction but also to the progression of liver injury [27]. Our findings provide mechanistic evidence linking Pema treatment with the regulation of leptin production. We demonstrated that insulin stimulation increased EGR-1 expression while suppressing Reg-1 in differentiated 3T3-L1 adipocyte-like cells, consistent with the established role of EGR-1 as a transcriptional regulator of insulin-dependent leptin expression [28,29]. Pema markedly inhibited insulin-induced EGR-1 expression while simultaneously inducing PPARα expression, resulting in a significant reduction in leptin secretion. These observations indicate that activation of PPARα suppresses leptin production, at least in part, through inhibition of the EGR-1 signaling pathway. Previous studies have shown that Pema suppresses inflammatory signaling through inhibition of NF-κB activation and subsequently reduces the expression of pro-inflammatory cytokines, including TNF-α, IL-6, and IL-1β [30,31,32]. Because NF-κB directly regulates EGR-1 transcription [33,34,35], inhibition of this pathway may explain the reduced EGR-1 expression observed in the present study. Although the precise molecular interactions require further investigation, our findings support a mechanistic model in which Pema suppresses inflammatory signaling upstream of EGR-1, thereby reducing leptin production in adipocytes. Specifically, PPARα activation by Pema may modulate Reg1, an RNase essential for controlling inflammatory transcripts, which in turn blunts the insulin-mediated induction of EGR-1. Because local leptin expression was not detected in the liver, our mechanistic investigations regarding the EGR-1, PPARα, and Reg1 regulatory axis were specifically focused on adipocytes. Therefore, the direct local effects of Pema on the expression of these molecules in hepatic cells were not evaluated in the present study. Importantly, the molecular findings observed in cultured adipocyte-like cells were consistently reproduced in vivo. In CDAHFD-fed mice, Pema reduced circulating insulin and leptin concentrations and markedly decreased hepatic accumulation of both proteins. In preliminary experiments, we also confirmed expression trends of EGR-1, Reg1, and PPARα in the visceral adipose tissue of CDAHFD-fed mice; however, these findings were exploratory and thus not included in the formal results. Moreover, patients with MASLD and hypertriglyceridemia who received long-term Pema therapy exhibited significantly lower serum leptin levels together with improvements in liver enzymes, surrogate markers of fibrosis, and insulin resistance. Although the reduction in serum leptin in this clinical cohort was modest, it reached statistical significance and was consistently accompanied by improvements in clinical parameters and experimental findings. The concordance among cellular, animal, and clinical findings strengthens the conclusion that modulation of leptin signaling represents an important therapeutic action of Pema. Collectively, these findings indicate that excessive leptin production may contribute to disease progression in MASLD and that suppression of leptin signaling represents a previously underrecognized mechanism through which SPPARMα exerts hepatoprotective effects. Beyond its established role in improving lipid metabolism, Pema appears to regulate adipose tissue–liver crosstalk, thereby providing broader metabolic benefits that may delay the progression of MASLD.

The beneficial effects of Pema may also extend beyond hepatic lipid metabolism through modulation of gut microbiota. As a selective PPARα modulator, Pema may influence the gut microbial composition through several potential pathways, including the modulation of host lipid metabolism, alterations in bile acid profiles, suppression of intestinal inflammation, and reinforcement of gut barrier function. In the present study, Pema treatment dose-dependently increased the abundance of the phylum *Verrucomicrobia* in CDAHFD-fed mice, suggesting that alterations in the intestinal microbial community may contribute to its hepatoprotective effects [36]. Among members of this phylum, *Akkermansia muciniphila* has attracted considerable attention because of its association with metabolic health and maintenance of intestinal barrier integrity. *A. muciniphila* is a mucin-degrading anaerobic bacterium that colonizes the intestinal mucus layer and is commonly detected in healthy individuals [37,38,39]. Clinical and experimental studies have consistently demonstrated reduced abundance of *A. muciniphila* in obesity, type 2 diabetes, and MASLD [40,41]. Restoration of this bacterium has been associated with improved metabolic homeostasis, enhanced intestinal barrier function, reduced systemic inflammation, and attenuation of hepatic steatosis. These beneficial effects are thought to be mediated, at least in part, through bacterial membrane proteins, extracellular vesicles, and modulation of host immune responses. Although the present study was not designed to determine whether the increase in the phylum *Verrucomicrobia* directly mediated the therapeutic effects of Pema or how these microbial changes mechanistically link to the observed leptin signaling suppression, our findings support the concept that remodeling of the gut microbiota may represent an additional mechanism contributing to disease improvement. Previous studies using dietary steatotic liver disease models have similarly reported favorable alterations in the intestinal microbiota following Pema administration, supporting the reproducibility of this observation [42]. Given the close interaction between the intestine and the liver through the gut–liver axis, microbiota remodeling may reduce intestinal permeability, suppress hepatic inflammation, and improve metabolic homeostasis. Further mechanistic studies integrating metagenomics, metabolomics, and functional microbiome analyses will be necessary to clarify the causal relationship between Pema-induced microbial alterations and improvements in MASLD.

Several limitations of this study should be acknowledged. First, liver biopsy specimens were not available for all patients before and after treatment. Although histological assessment remains the reference standard for evaluating MASH, liver biopsy is invasive and subject to sampling variability and interobserver differences. Therefore, validated non-invasive biomarkers and imaging-based assessments, including those used in the present study, represent practical alternatives for longitudinal clinical evaluation. Second, the clinical portion of this research was a retrospective observational study lacking a placebo or active comparator arm for efficacy control. Additionally, this was a relatively small observational study conducted exclusively in Japanese institutions, which may limit the generalizability of our findings to other ethnic populations. Finally, although our experimental results strongly suggest that suppression of leptin signaling contributes to the therapeutic effects of Pema, this evidence remains associative. A definitive causal link—confirming whether leptin reduction is the primary driver of the observed hepatic amelioration—has not been fully established in the present study. Future investigations utilizing genetic or pharmacological interventions, such as leptin supplementation or receptor-blocking models, will be necessary to definitively validate the causal relationships among PPARα activation, EGR-1 inhibition, leptin regulation, and gut microbiota remodeling. While our findings specifically highlight the novel contribution of leptin signaling and gut microbiota to the therapeutic effects of Pema, the present study did not evaluate its impact on classical lipid synthesis pathways, such as the hepatic expression of SREBPs. Given the central role of SREBPs in lipid homeostasis and their known crosstalk with PPARα, future studies integrating the SREBP regulatory axis with our leptin-microbiota findings will be necessary to fully decode the comprehensive lipid-lowering actions of Pema.

In summary, our findings demonstrate that long-term Pema treatment improves liver-related biochemical parameters, surrogate markers of hepatic fibrosis, insulin resistance, and circulating leptin levels in patients with MASLD. Complementary cellular and animal experiments further indicate that these beneficial effects are associated with suppression of EGR-1-dependent leptin production, attenuation of hepatic inflammation and fibrosis, and favorable remodeling of the gut microbiota. Collectively, these results identify leptin signaling and the gut–liver axis as complementary therapeutic targets of SPPARMα and provide mechanistic evidence supporting the repositioning of Pema as a promising therapeutic strategy for MASLD accompanied by hypertriglyceridemia. Future prospective multicenter studies and mechanistic investigations will help define its clinical utility and optimize its role in the management of MASLD.

## 4. Materials and Methods

### 4.1. Animals and Experimental Model

Male C57BL/6J mice, aged five weeks, were sourced from Japan SLC, Inc. (Shizuoka, Japan) and subjected to a one-week acclimatization period prior to the experiments. All animals were maintained in a temperature- and humidity-controlled environment under a standard 12-h light/dark cycle, with ad libitum access to regular drinking water.

For diet interventions, the control group was fed a standard diet (SD; 3.6 kcal/g, containing 51 g/kg crude fat). Experimental models of MASLD were induced using a choline-deficient, L-amino acid-defined, high-fat diet (CDAHFD; Cat. #A06071302, Research Diets, Inc., New Brunswick, NJ, USA), which supplied 5.2 kcal/g with 60% of calories derived from fat and 0.1% methionine. Consistent with previous literature, this dietary regimen successfully mimics the full spectrum of MASLD pathology in C57BL/6J mice over a 12-week timeframe [34,41]. Pemafibrate (Pema), provided by Kowa Co., Ltd. (Nagoya, Japan), was prepared in a 0.5% methylcellulose vehicle (MC; Cat. #133-17815, Wako Pure Chemical Corporation, Osaka, Japan). Mice in the treatment arms received daily doses of either 0.1 or 1 mg/kg Pema via oral gavage using a sonde (KN-348-20G-70, Natsume Seisakusho Co., Ltd., Tokyo, Japan), while the vehicle control group was administered 0.5% MC alone (Figure 1a). Body weights were recorded on a weekly basis. At the study’s conclusion, euthanasia was carried out via exsanguination under isoflurane anesthesia. Blood was drawn from the postcaval vein, and serum fractions were stored at −80 °C for subsequent biochemical profiling. Liver tissues were rapidly excised; fragments intended for molecular assays were snap-frozen in liquid nitrogen or on dry ice, whereas sections for histological evaluation were immediately immersed in 10% neutral-buffered formalin. Fresh fecal pellets were gathered from each animal and cryopreserved at −80 °C.

### 4.2. Biochemical Measurements

Circulating murine leptin (Cat. #ab100718, Abcam K.K., Tokyo, Japan) and insulin (Cat. #ab277390, Abcam K.K.) were quantified utilizing sandwich enzyme-linked immunosorbent assay (ELISA) kits. The clinical assessment of human serum leptin, alongside the biochemical profiling of mouse hepatic lipid composition, was outsourced to SRL Central Laboratory (Tokyo, Japan). Human leptin data were derived from 95 clinical subjects who possessed adequately preserved baseline serum aliquots.

### 4.3. Fecal Microbiome Profiling

Genomic profiling of the fecal microbiome via 16S rDNA sequencing was executed by Bioengineering Lab. Co. Ltd. (Kanagawa, Japan), adhering to protocols detailed in our earlier work [36].

### 4.4. Histological and Immunohistochemical Assessment

Following formalin fixation, hepatic tissues were processed into paraffin blocks. These blocks were sectioned at a thickness of 5 μm and subsequently subjected to hematoxylin and eosin (H&E) as well as Sirius Red staining protocols. Digital archiving and pathological review of the slides were facilitated by a NanoZoomer Digital Pathology scanner (Hamamatsu Photonics K.K., Shizuoka, Japan). Morphometric quantification of steatotic and fibrotic regions based on Sirius Red staining was performed using ImageJ software (v1.49; National Institutes of Health, Bethesda, MD, USA). The extent of fibrosis and steatosis was expressed as a percentage of the total cross-sectional tissue area. For each specimen, calculations were averaged from seven independent fields of view randomly captured at 10× magnification (n = 4 animals per experimental condition). For immunohistochemistry, the 5 μm paraffin slices were probed using primary monoclonal antibodies targeting insulin (Cat. #ab181547, diluted 1:5000; Abcam) and leptin (Cat. #ab16227, diluted 1:50; Abcam K.K., Tokyo, Japan).

### 4.5. RNA Extraction and Quantitative Real-Time PCR

Hepatic total RNA was isolated utilizing the RNeasy Mini Kit (Qiagen, Tokyo, Japan). Reverse transcription into cDNA was accomplished with the High-Capacity cDNA Reverse Transcription Kit (Applied Biosystems, Foster City, CA, USA).

Quantitative PCR (qPCR) assays were run on a StepOnePlus Real-Time PCR System (Applied Biosystems). The thermal cycling profile consisted of an initial denaturation step at 95 °C for 20 s, followed by 40 amplification cycles (95 °C for 1 s, then 60 °C for 20 s). Transcript levels were quantified employing TaqMan Gene Expression Assays with custom-designed primers and probes (Applied Biosystems). Data were normalized against the endogenous control, rodent GAPDH (Life Technologies Japan, Tokyo, Japan), and relative mRNA expression fold changes were determined via the standard curve methodology.

### 4.6. Cell Culture and Immunoblotting

The murine 3T3-L1 preadipocyte cell line, acquired from the Japanese Collection of Research Bioresources Cell Bank, was cultured in Dulbecco’s Modified Eagle Medium containing 10% fetal bovine serum. Differentiation into a mature adipocyte-like phenotype was induced over 7 to 10 days utilizing an adipogenic cocktail consisting of 50 mM 3-isobutyl-1-methylxanthine (Cat. #095-03413, Wako Pure Chemical Corporation), 10 μg/mL insulin (Cat. #096-06483, Wako), and 1 mM dexamethasone (Cat. #096-06483, Wako). Mature adipocyte-like cells were then exposed to 1 μg/mL insulin and 1 μg/mL Pema for a 24-h period prior to cellular harvest. Extracted protein lysates underwent separation via sodium dodecyl sulfate–polyacrylamide gel electrophoresis (SDS-PAGE) and subsequent immunoblot analysis, according to our previously published protocols [42]. Immunoblotting involved the following primary monoclonal antibodies (mAbs): anti-EGR-1 (clone 44D5, Cell Signaling Technology, Tokyo, Japan), anti-PPARα (Cat. #ab126285, Abcam K.K.), and anti-regnase-1 (Cat. #MABF936, Merck Life Science Ltd., Tokyo, Japan). Beta-actin (clone AC-15, Merck Life Science) served as the internal loading control. Horseradish peroxidase (HRP)-conjugated secondary antibodies directed against mouse (GE Healthcare, Tokyo, Japan) and rabbit (Agilent Technologies Japan, Ltd., Tokyo, Japan) IgG were utilized for signal detection.

### 4.7. Study Participants

The clinical cohort consisted of 192 subjects diagnosed with hypertriglyceridemia, recruited from Hokkaido University Hospital and JCHO Hokkaido Hospital during the period from July 2021 to March 2023. The diagnostic criterion for MASLD was the ultrasonic confirmation of hepatic steatosis. This diagnosis necessitated the exclusion of other etiologies, including excessive alcohol intake (exceeding 210 g/week for males or 140 g/week for females), drug-induced fatty liver, chronic viral hepatitis (HBV or HCV), and the histological presence of alternative chronic hepatopathies. Additional exclusion criteria comprised a history of bariatric surgery, chronic inflammatory bowel disease, active systemic infections, hepatocellular carcinoma, or decompensated cirrhotic events such as ascites or esophageal variceal hemorrhage.

Data for patient demographic characteristics (age, sex, daily dosage of Pema, concomitant disease, and BMI), liver enzyme levels (AST, ALT, and GGT), lipid parameters (triglyceride, LDL cholesterol, and HDL cholesterol levels), liver fibrosis markers (platelet count, M2BPGi, and FIB–4 index), and glucose metabolism parameters (HbA1c and HOMA–IR) were collected every 1–3 months from medical records. Transient elastography (FibroScan; Echosens, Paris, France) was performed at baseline and weeks 48 and 96 to obtain LSM and CAP, according to our previous study protocol [36].

### 4.8. Statistical Analysis

Continuous variables are expressed as the mean ± standard deviation (SD). Group comparisons were executed utilizing Student’s t-test or analysis of variance (ANOVA), as appropriate. For categorical variables, the Chi-squared test or Fisher’s exact test was applied. Non-parametric numerical data were evaluated with the Wilcoxon rank-sum test. All statistical computations were conducted via JMP Pro software, version 17.2.0 (SAS Institute Inc., Cary, NC, USA), with a *p*-value threshold of <0.05 defining statistical significance.

## Figures and Tables

**Table 1:** Characteristics of 192 patients with steatotic liver disease and hypertriglyceridemia at baseline and 1 and 2 years after pemafibrate treatment.

n = 192	Baseline	1 Year	*p* Value vs. Baseline	2 Years	*p* Value vs. Baseline
Age (years)	59 (22−85)				
Male sex (%)	139 (72.4)				
Daily dosage of pemafibrate 0.2 mg or 0.4 mg	108 or 84				
Type 2 diabetes mellitus (%)	54 (28.1)				
Hypertension (%)	64 (33.2)				
BMI (kg/m^2^)	26.8 (18.6−42.2)	26.3 (17.5−37.6)	n.s.	26.6 (18.6−38.4)	n.s.
Platelet count (10^10^/L)	21.4 (7.8−43.2)	22.8 (7.8−46.1)	n.s.	23.6 (6.8−43.8)	0.02 *
AST (IU/L)	37 (13−167)	30 (15−112)	<0.01 *	26 (13−88)	<0.01 *
ALT (IU/L)	49 (9−196)	30 (7−115)	<0.01 *	25 (5−81)	<0.01 *
GGT (IU/L)	67 (18−554)	33 (10−322)	<0.01 *	30 (11−414)	<0.01 *
Triglyceride (mg/dL)	150 (54−1296)	85.5 (33−297)	<0.01 *	91 (38−301)	<0.01 *
LDL cholesterol (mg/dL)	119.5 (33−264)	100.5 (47−176)	<0.01 *	102 (41−167)	<0.01 *
HDL cholesterol (mg/dL)	47 (27−93)	47 (25−101)	n.s.	47 (25−97)	n.s.
HbA1c (%)	6.0 (4.4−9.8)	6.1 (4.7−8.9)	n.s.	6.0 (4.6−8.2)	n.s.
HOMA-IR	3.3 (1.7−5.3)	3.0 (1.4−4.6)	n.s.	2.7 (1.4−4.3)	0.04 *
M2BPGi (COI)	0.9 (0.3−6.1)	0.6 (0.1−7.9)	<0.01 *	0.6 (0.2−3.0)	<0.01 *
FIB-4 index	1.45 (0.11−7.06)	1.59 (0.31−8.40)	n.s.	1.37 (0.31−5.45)	0.04 *
LSM (kPa)	6.2 (3.0−51.1)	6.3 (2.7−43.3)	n.s.	5.3 (3.1−40.0)	0.01 *
CAP (dB/m)	299 (100−400)	308 (100−624)	n.s.	295 (100−400)	n.s.
Leptin (ng/mL) ^a^	9.1 (1.5−47.7)			8.9 (0.7−40.1)	0.02 *

Note: Values are expressed as the median (range). * Statistical significance was set at a *p* value of <0.05. Abbreviations: ALT, alanine aminotransferase; AST, aspartate aminotransferase; BMI, body mass index; CAP, controlled attenuated parameter; FIB-4, fibrosis-4; GGT, γ-glutamyl transferase; HbA1c, glycated hemoglobin; HDL, high-density lipoprotein; HOMA–IR, homeostasis model assessment as an index of insulin resistance; LDL, low-density lipoprotein; LSM, liver stiffness measurement; M2BPGi, Wisteria floribunda agglutinin-positive Mac-2 binding protein glycan isomer; n.s., not significant. ^a^ The data include only 95 patients whose serum leptin levels were available.

**Table 2:** Correlation analyses between serum leptin levels and clinical parameters.

n = 95	vs. Baseline Leptin		vs. Delta Leptin	
	r	*p* Value	r	*p* Value
BMI (kg/m^2^)	0.262	0.01 *	0.021	n.s.
Platelet count (10^10^/L)	0.139	n.s.	−0.020	n.s.
AST (IU/L)	0.265	<0.01 *	−0.256	0.01 *
ALT (IU/L)	0.252	0.01 *	−0.224	0.03 *
GGT (IU/L)	0.034	n.s.	−0.161	n.s.
Triglyceride (mg/dL)	−0.125	n.s.	−0.017	n.s.
LDL cholesterol (mg/dL)	−0.013	n.s.	−0.079	n.s.
HDL cholesterol (mg/dL)	0.256	0.01 *	−0.206	n.s.
HbA1c (%)	0.023	n.s.	−0.101	0.04 *
HOMA-IR	0.393	<0.01 *	−0.282	0.01 *
M2BPGi (COI)	0.029	n.s.	−0.049	n.s.
FIB-4 index	−0.037	n.s.	−0.073	n.s.
LSM (kPa)	0.123	n.s.	−0.152	n.s.
CAP (dB/m)	0.078	n.s.	0.063	n.s.

Note: * Statistical significance was set at a *p* value of <0.05. Delta leptin was calculated by subtraction (serum leptin at 2 years minus baseline). Abbreviations: ALT, alanine aminotransferase; AST, aspartate aminotransferase; BMI, body mass index; CAP, controlled attenuated parameter; FIB-4, fibrosis-4; GGT, γ-glutamyl transferase; HbA1c, glycated hemoglobin; HDL, high-density lipoprotein; HOMA–IR, homeostasis model assessment as an index of insulin resistance; LDL, low-density lipoprotein; LSM, liver stiffness measurement; M2BPGi, Wisteria floribunda ag-glutinin-positive Mac-2 binding protein glycan isomer; n.s., not significant. The data include only 95 patients whose serum leptin levels were available.

## Data Availability

The data presented in this study are available from the corresponding author upon reasonable request. The data are not publicly available because they contain information that could compromise the privacy of research participants.

## Supplementary Materials

The following supporting information can be downloaded at: https://www.mdpi.com/article/10.3390/ijms27177611/s1.

## Abbreviations

Alb, albumin; ALT, alanine aminotransferase; αSMA, α smooth muscle actin; AST, aspartate aminotransferase; BMI, body mass index; CDAHFD, choline-deficient, L-amino acid-defined, high-fat diet; Col1A1, collagen 1A1; DM, diabetes mellitus; EGR-1, early growth response protein 1; GGT, γ glutamyl transferase; GAPDH, glyceraldehyde 3-phosphate dehydrogenase; H&E, hematoxylin and eosin; HBP, high blood pressure; HOMA–IR, homeostasis model assessment as an index of insulin resistance; LC, liver cirrhosis; LDL, low-density lipoprotein; M2BPGi, *Wisteria floribunda* agglutinin-positive Mac-2 binding protein glycan isomer; MASLD, metabolic dysfunction-associated steatotic liver disease; NAFLD, nonalcoholic fatty liver disease; NASH, nonalcoholic steatohepatitis; n.s., not significant; Pema, pemafibrate; PPAR, peroxisome proliferator-activated receptor; Reg1, regnase-1; Plt, platelet count; SPPARMα, selective PPARα modulator; SDS-PAGE, sodium dodecyl sulfate–polyacrylamide gel electrophoresis; SD, standard diet; SLD, steatotic liver disease; TLR4, Toll-like receptor 4; TGFβ, transforming growth factor β; TG, triglyceride; TNFα, tumor necrosis factor α.
