# Supplementary material for: Selective PPARα Modulator Pemafibrate Ameliorates Metabolic Dysfunction-Associated Steatotic Liver Disease Through Regulation of Leptin Signaling and Remodeling of Gut Microbiota
Source: Int J Mol Sci. 2026 Aug 25;27(17):7611. doi: 10.3390/ijms27177611 (PMC13566180; doi:10.3390/ijms27177611)
Supplement: Supplementary file 1 [file ijms-27-07611-s001.zip › ijms-4472447-supplementary.pdf]

Table S1. Composition of the gut microbiome (phylum) of mice fed with SD and CDAHFD (n=8 per group) by administration of Pema at 12 weeks.

Diet	SD	CDAHFD	*P* value vs. SD	CDAHFD	*P* value vs. SD	CDAHFD	*P* value vs. SD
Dosage of Pema (mg/kg/day)	0	0		0.1		1.0	
phylum Others	0.0 (0.0–0.0)	0.0 (0.0–0.3)	n.s.	0.0 (0.0–1.2)	n.s.	0.0 (0.0–0.0)	n.s.
phylum *Deferribacteres*	1.1 (0.7–1.2)	0.6 (0.0–1.6)	n.s.	1.0 (1.0–1.5)	n.s.	0.5 (0.2–0.6)	n.s.
phylum *Verrucomicrobia*	0.0 (0.0–0.0)	4.0 (4.0–4.6)	<0.01*	13.9 (9.2–22.4)	<0.01*	21.0 (6.5–25.0)	<0.01*
phylum *Proteobacteria*	0.0 (0.0–0.0)	0.7 (0.3–1.3)	n.s.	0.4 (0.2–0.7)	n.s.	0.6 (0.4–3.71)	n.s.
phylum *Actinobacteria*	2.4 (2.2–3.6)	5.5 (1.7–8.0)	n.s.	4.1 (3.5–6.7)	n.s.	4.3 (2.6–7.8)	n.s.
phylum *Bacteroidetes*	26.7 (23.2–34.7)	2.0 (1.3–4.4)	<0.01*	2.7 (1.3–6.7)	0.03*	3.3 (3.0–4.4)	0.02*
phylum *Firmicutes*	69.9 (61.8–72.5)	86.3 (85.7–87.8)	<0.01*	75.8 (66.0–81.8)	n.s.	67.3 (66.9–82.4)	n.s.

Note: 0.C. DAHFD, choline-deficient, L-amino acid-defined, high fat diet; Pema, pemafibrate; SD, standard diet.
